# Ein- oder zweizeitiges Vorgehen nach Osteosyntheseversagen bei proximaler Femurfraktur – ein narrativer Review

**DOI:** 10.1007/s00132-025-04747-z

**Published:** 2025-12-05

**Authors:** Christina Pempe, Suzanne Zeidler, Andreas Roth

**Affiliations:** Universitätsklinikum Leipzig AöR, Liebigstr. 20, 04103 Leipzig, Deutschland

**Keywords:** Geriatrie, Infektion, Gelenkprothese, Revision Gelenk, Hüfttotalendoprothese, Geriatrics, Infection, Joint protheses, Revision joint, Total hip arthroplasty

## Abstract

**Hintergrund:**

Das Osteosyntheseversagen nach proximaler Femurfraktur stellt eine zunehmende Herausforderung in der Alterstraumatologie dar. In diesen Fällen ist häufig eine Konversions-Hüfttotalendoprothese (K-HTEP) erforderlich. Unklar ist, ob ein einzeitiges oder zweizeitiges Vorgehen hinsichtlich Infektionsrisiko und klinischem Outcome überlegen ist.

**Methodik:**

Es wurde eine Literaturrecherche in der Datenbank PubMed für den Zeitraum 1995–2025 durchgeführt. Eingeschlossen wurden Originalarbeiten, die das ein- oder zweizeitige Vorgehen nach Osteosyntheseversagen des proximalen Femurs untersuchten. Fallberichte, Editorials und Studien ohne Bezug zur Hüftendoprothetik wurden ausgeschlossen. Insgesamt wurden 17 Publikationen in die Analyse eingeschlossen. Auf Grundlage der Ergebnisse wurde ein klinischer Entscheidungsalgorithmus zur Therapieplanung in der eigenen Klinik entwickelt.

**Ergebnisse:**

Die verfügbare Evidenz basiert überwiegend auf retrospektiven Studien mit geringer Fallzahl. Infektionsraten nach K‑HTEP variieren erheblich zwischen den Studien (0–8,6 %). Einzelne Arbeiten zeigen Vorteile des zweizeitigen Vorgehens hinsichtlich Infektionskontrolle, während andere vergleichbare Ergebnisse für beide Strategien aufzeigen. Positive mikrobiologische Befunde trotz fehlender klinischer Infektzeichen sind häufig, ihre klinische Relevanz bleibt unklar.

**Schlussfolgerung:**

Die aktuelle Datenlage erlaubt keine eindeutige Empfehlung zugunsten eines ein- oder zweizeitigen Vorgehens. Entscheidend ist eine individuelle Risikostratifizierung unter Berücksichtigung klinischer, laborchemischer und radiologischer Parameter. Der vorgestellte klinische Algorithmus bietet einen praxisorientierten Ansatz zur Entscheidungsfindung. Prospektive, multizentrische Studien sind erforderlich, um belastbare Empfehlungen abzuleiten.

## Abstract (en)

**Background:**

Failure of osteosynthesis following proximal femur fracture represents an increasing challenge in geriatric traumatology. In these cases, conversion total hip arthroplasty (cTHA) is often required. It remains unclear whether a single-stage or a two-stage approach is superior in terms of infection risk and clinical outcomes.

**Methods:**

A systematic literature search was conducted in PubMed for the period 1995–2025. Included were original articles, systematic reviews, and meta-analyses investigating single-stage or two-stage procedures following osteosynthesis failure of the proximal femur. Case reports, editorials, and studies unrelated to hip arthroplasty were excluded. A total of 17 publications were included in the analysis. Based on the findings, a clinical decision-making algorithm for treatment planning was developed in our own clinic.

**Results:**

The available evidence is predominantly based on retrospective studies with small sample sizes. Reported infection rates after cTHA vary widely between studies (0–8.6%). Some studies indicate advantages of the two-stage approach in terms of infection control, while others report comparable outcomes for both strategies. Positive microbiological findings despite the absence of clinical signs of infection are common, but their clinical relevance remains unclear.

**Conclusion:**

Current evidence does not allow a definitive recommendation in favor of either a single-stage or two-stage approach. Individual risk stratification, considering clinical, laboratory, and radiological parameters, is crucial. The clinical algorithm presented provides a practical approach for decision-making. Prospective multicenter studies are needed to derive robust recommendations.

## Einleitung

Proximale Femurfrakturen sind mit rund 23 % die häufigste Fraktur beim Erwachsenen und stellen eine wachsende Herausforderung in der Alterstraumatologie dar [1]. Durch die demografische Entwicklung ist in den kommenden Jahren mit einem weiteren Anstieg zu rechnen. Trotz moderner Implantate und Osteosyntheseverfahren benötigen bis zu 14 % der Patienten eine Revisionsoperation [2, 3]. Ursachen sind die sekundäre Koxarthrose, die Femurkopfnekrose, ausbleibende Frakturheilung sowie Osteosyntheseversagen mit sekundärer Dislokation oder Cut-Out [3–5]. In diesen Fällen ist häufig ein endoprothetischer Gelenkersatz notwendig, welcher auch als Konversions-Hüfttotalendoprothese bezeichnet wird.

Die K‑HTEP unterscheidet sich in wesentlichen Punkten von der primären Hüfttotalendoprothese. Studien berichten über signifikant längere Operationszeiten, einen längeren Krankenhausaufenthalt sowie höhere Behandlungskosten [6, 7]. Defektsituationen und Substanzverluste machen häufiger den Einsatz von Revisionsimplantaten erforderlich [7–9]. Zusätzlich gehen K‑HTEP mit einem höheren intraoperativen Blutverlust sowie schlechteren postoperativen Ergebnissen einher [8, 10]. Komplikationen wie Luxationen, periprothetische Frakturen oder Infektionen treten danach signifikant häufiger auf [11]. In vielen Aspekten ähnelt die K‑HTEP daher einer Revisionsendoprothese und wird in der Literatur häufig als „revision light“ bezeichnet [12, 13]. Ein zusätzlicher Aspekt ist der Zeitpunkt der Konversion. Grayson et al. berichteten über einen Trend zu höheren Komplikationsraten bei „früher“ Konversion, innerhalb von 6 Monaten nach Osteosynthese am Femur oder Azetabulum, insbesondere im Hinblick auf periprothetische Infektionen und Revisionsoperationen [14]. Auch Tate et al. beobachteten bei Konversionen innerhalb eines Jahres signifikant erhöhte Komplikations‑, Infektions- und Reoperationsraten [15]. Ergänzend zeigten Magnuson et al. eine Zunahme an medizinischen Komplikationen und Wiederaufnahmeraten [16]. Dieser mögliche „second hit“ sollte daher im klinischen Alltag besondere Beachtung finden.

## Methodik der Literaturrecherche

Im Rahmen dieser narrativen Übersicht wurde eine Literaturrecherche in der Datenbank PubMed (National Library of Medicine, USA) durchgeführt. Ziel war die Identifikation relevanter Studien zum ein- und zweizeitigen Vorgehen nach Osteosyntheseversagen bei proximaler Femurfraktur sowie zur K‑HTEP, insbesondere hinsichtlich Infektionsrisiken und mikrobiologischer Diagnostik.

Als Suchbegriffe dienten: „conversion total hip arthroplasty“, „failed internal fixation“, „proximal femur fracture“, „hardware removal“, „single-stage“, „two-stage“ und „periprosthetic joint infection“. Diese Begriffe wurden mit AND und OR kombiniert. Der Suchzeitraum umfasste Publikationen von 1995 bis 2025, um sowohl aktuelle als auch historisch relevante Arbeiten zu erfassen. Eingeschlossen wurden Originalarbeiten, systematische Reviews und Metaanalysen in Peer-reviewten Fachzeitschriften. Ausschlusskriterien waren Fallberichte mit weniger als fünf Fällen, Abstracts, Editorials sowie Studien ohne direkten Bezug zur Hüftendoprothetik oder nach anderen fehlgeschlagenen Osteosynthesen.

Die Auswahl erfolgte in mehreren Schritten. Zunächst wurden Titel und Abstracts gesichtet. Anschließend wurden die Volltexte relevanter Arbeiten analysiert. Insgesamt konnten 66 Studien in die weitere Analyse aufgenommen werden. Nach Ausschluss von Arbeiten ohne Bezug zu mikrobiologischen Befunden, periprothetischer Infektionen oder abweichender Auswertungen verblieben 17 Studien, die die aktuelle Evidenz zum ein- und zweizeitigen Vorgehen nach Osteosyntheseversagen widerspiegeln. Sie dienten als Grundlage für einen klinischen Entscheidungsalgorithmus in unserer Klinik.

## Studienlage und methodische Bewertung

Die Daten zum ein- versus zweizeitigen Vorgehen nach Osteosyntheseversagen proximaler Femurfrakturen stammen überwiegend aus retrospektiven Fallserien und Vergleichsstudien mit kleiner Fallzahl. Prospektive oder randomisierte Studien liegen bislang nicht vor. Entsprechend liegt das Evidenzniveau der meisten Arbeiten zwischen Level III und IV (Oxford Centre for Evidence-Based Medicine). Die Studien unterscheiden sich deutlich bezüglich Patientenselektion, Definition von Infektionsparametern und diagnostischen Standards (z. B. Anzahl entnommener Gewebeproben, Sonikation, PCR). Wichtige Bias-Risiken ergeben sich durch retrospektives Design, heterogene Patientenkohorten, fehlende Kontrollgruppen und heterogene Nachbeobachtungszeiträume (6 Monate bis > 2 Jahre). Dadurch ist das Risiko für Selektions- oder Informationsbias erhöht und erschwert die Vergleichbarkeit der Ergebnisse.

Einige Arbeiten [8, 17] zeigen, dass ein einzeitiges Vorgehen unter strenger Indikationsstellung sicher sein kann. Andere [18, 19] berichten über höhere Infektionsraten und empfehlen ein zweizeitiges Vorgehen. Die Diskrepanz der Ergebnisse verdeutlicht die methodischen Schwächen der Evidenzbasis. Ein limitierender Faktor ist die geringe Fallzahl sowie das Fehlen standardisierter Kriterien zur Diagnostik und Infektbeurteilung.

Aufgrund dieser methodischen Limitationen ist die Ableitung eindeutiger Empfehlungen für ein ein- oder zweizeitiges Vorgehen derzeit nicht möglich. Zur besseren Übersicht sind die wichtigsten Studien, ihre Evidenzlevel und methodischen Limitationen in Tab. 1 zusammengefasst.

**Tab. 1:** Übersicht der wichtigsten Studien zum ein- versus zweizeitigen Vorgehen nach Osteosyntheseversagen proximaler Femurfrakturen.

Studie	Jahr	Design/Fallzahl	Vorgehen (ein-/zweizeitig)	Outcome	Evidenz-Level	Methodische Limitationen
Klatte et al.	[17]	Retrospektiv *n* = 122	Einzeitige K‑HTEP vs. primäre HTEP	Präoperative Gelenkaspiration: 1,9 % (1/52) positiv; intraoperative Proben: 0,9 % (1/109) positiv; keine PJI beobachtet	IV	Retrospektiv, selektierte Patientengruppe, unvollständige mikrobiologische Daten
Hernandez et al.	[5]	Retrospektiv, *n* = 62	Einzeitige K‑HTEP	PJI-Rate: 1,6 % (1/62)	IV	Retrospektiv, keine Vergleichsgruppe, selektierte Kohorte, nur Patienten nach Schrauben-osteosynthese
George et al.	[20]	Retrospektiv *n* = 41	Einzeitige K‑HTEP	51 % positive mikrobiologische Befunde; 12 % postoperative Komplikationen	IV	Retrospektiv, Kleine Fallzahl, keine klinische Korrelation
Scholten et al.	[19]	Retrospektiv *n* = 187	Ein- vs. zweizeitige K‑HTEP	Höhere PJI-Rate nach einzeitigem Vorgehen (8,6 % vs. 3,8 %)	III	Retrospektiv, mögliche Selektionsbias, unklare Definition PJI
Smith et al.	[21]	Retrospektiv, *n* = 369	Einzeitige K‑HTEP vs. primäre HTEP	Höhere PJI-Rate nach K‑HTEP als nach primärer HTEP (6,2 % vs. 2,6 %)	III	Retrospektiv, nur Patienten nach Marknagelosteosynthese
Palmowski et al.	[22]	Retrospektiv, *n* = 32	Ein- und zweizeitige K‑HTEP	Positive mikrobiologische Befunde: 15,6 % (5/32); PJI-Rate: 6,2 % (2/32) nach einzeitiger K‑HTEP	IV	Retrospektiv, kleine Fallzahl, heterogene Patientenkohorte
Corradi et al.	[23]	Retrospektiv, *n* = 14	Einzeitige K‑HTEP	PJI-Rate: 7,1 % (1/14)	IV	Retrospektiv, sehr kleine Fallzahl, keine Vergleichsgruppe
Hemmann et al.	[24]	Retrospektiv *n* = 52	Einzeitige K‑HTEP	Positiver Erregernachweis: 10 % (7/71); PJI-Rate: 5,8 % (4/71)	IV	Retrospektiv, Kleine Fallzahl, keine Vergleichsgruppe, heterogene Patientengruppe
Anderson et al.	[18]	Retrospektiv *n* = 71	Ein- vs. zweizeitige K‑HTEP	PJI-Rate (4,2 %) nur bei zweizeitiger K‑HTEP	III	Retrospektiv, ungleiche Gruppengröße
La Camera et al.	[8]	Retrospektiv, matched-pair *n* = 127 hips	Einzeitige K‑HTEP vs. primäre HTEP	Keine diagnostizierte PJI in beiden Gruppen	III	Retrospektiv, keine mikrobiologischen Daten, keine Angaben zum „loss to follow-up“
Sequeira et al.	[25]	Retrospektiv, matched-pair Analyse *n* = 5504	Ein- vs. zweizeitige K‑HTEP	PJI-Rate nach 90 Tagen niedriger bei einzeitigem Vorgehen (1,85 % vs. 3,05 %); PJI-Rate nach 1 Jahr niedriger bei einzeitigem Vorgehen (2,94 % vs. 4,14 %)	III	Retrospektiv, Follow-up über Krankenkassendaten, fehlende klinische Details
Solarino et al.	[26]	Retrospektiv, *n* = 74	Einzeitige K‑HTEP	PJI-Rate: 4 % (3/74)	IV	Retrospektiv, keine Vergleichsgruppe
Habib et al.	[27]	Retrospektiv, *n* = 71	Einzeitige K‑HTEP	PJI-Rate: 21,13 % (15/71)	IV	Retrospektiv, keine Angaben zu mikrobiologischen Befunden
Moreira et al.	[28]	Retrospektiv *n* = 101	Einzeitige K‑HTEP	Positive mikrobiologische Befunde: 5 % (5/101), davon keine beobachtete PJI	IV	Retrospektiv, Keine Vergleichsgruppe, Follow-up nur perioperativ
Di Martino et al.	[29]	Systematisches Review, 12 retrospektive Studien, *n* = 1260	K‑HTEP nach fehlgeschlagener Osteosynthese	PJI: 3,41 %	III	Heterogene Studien, kein direkter Vergleich zwischen ein- vs. zweizeitigem Vorgehen
Muffly et al.	[30]	Retrospektiv *n* = 60	Einzeitige K‑HTEP Vergleich des Zugangsweges	Keine diagnostizierte PJI	III	Retrospektiv, kleine Fallzahl, keine Angaben zu mikrobiologischen Befunden
Yin et al.	[31]	Metaanalyse, 6 retrospektive Studien *n* = 1301	K‑HTEP vs. primäre HTEP	PJI-Rate bei K‑HTEP signifikant höher als bei primärer HTEP	II–III	Heterogene Studienpopulation, begrenzte Anzahl an eingeschlossenen Studien, retrospektives Studiendesign

Angegeben sind Studiendesign, Fallzahl, Vorgehensweise, Outcome, Evidenzlevel (nach Oxford Centre for Evidence-Based Medicine) und methodische Limitationen

*PJI* „periprosthetic joint infection“, *K‑HTEP* Konversions-Hüfttotalendoprothese, *HTEP* Hüfttotalendoprothese

## Konversions-HTEP – operative Herausforderungen

Die operative Situation nach Versagen der Osteosynthese ist durch mehrere Faktoren erschwert. Die vormals bestehende Fraktur sowie die einliegenden Implantate führen häufig zu Substanzverlusten, Kortikalisdefekten und bei Fehlverheilung auch zu Deformierungen. Das geriatrische Alterskollektiv bedingt eine reduzierte Knochenqualität, insbesondere am Femur. Schraubenlöcher oder alte Marknagelkanäle erfordern eine individuelle Implantatwahl, häufig kommen daher metaphysär/diaphysäre oder diaphysäre Hüftschäfte zum Einsatz [7]. Weichteilkompromittierungen nach vorangegangenen Eingriffen und das damit verbundene erhöhte Infektions- und Operationsrisiko stellen zusätzliche Herausforderungen für die Planung dar [24]. Ergänzend beeinflussen Patientenfaktoren wie höheres Lebensalter, Multimorbidität und eine eingeschränkte Regenerationsfähigkeit die perioperative Strategie. Die K‑HTEP kann dabei sowohl als einzeitiges oder als zwei-/mehrzeitiges Verfahren erfolgen.

## Ein- versus zweizeitiges Vorgehen

Das einzeitige Vorgehen umfasst die Entfernung des Osteosynthesematerials und die Implantation einer Hüft-TEP im gleichen Eingriff. Die Vorteile liegen in der Vermeidung zusätzlicher Weichteilschädigungen, einem kürzeren Krankenhausaufenthalt und einer schnelleren Rekonvaleszenz. Zudem entfällt die Phase der Immobilisation, die insbesondere bei multimorbiden Patienten mit erheblichen Risiken verbunden ist [24]. Nachteilig ist die längere Operationsdauer mit erhöhtem Blutungsrisiko [32]. Zudem müssen Revisionsimplantate vorgehalten werden, um auf intraoperative Defektsituationen nach Entfernung des Osteosynthesematerials individuell reagieren zu können. Nicht zuletzt kann eine Infektion präoperativ nicht mit Sicherheit ausgeschlossen werden.

Beim zweizeitigen Vorgehen wird zunächst das Osteosynthesematerial entfernt; gegebenenfalls erfolgt auch die Entfernung des Hüftkopfes und die Einlage eines Abstandshalters. Dadurch ist ein präoperativer Infektausschluss sicherer möglich, und eine bestehende Infektion kann zunächst behandelt werden. Die definitive Implantation der Hüft-TEP erfolgt erst in einem weiteren Eingriff. Dieses Vorgehen bietet ein höheres Maß an Sicherheit hinsichtlich der Infektkontrolle und ermöglicht zudem eine optimale Planung des zweiten Eingriffs. Nachteilig sind die höhere Anzahl an Operationen, was insbesondere für ältere und multimorbide Patienten eine erhebliche Belastung darstellen kann, sowie eine längere Rekonvaleszenz, Immobilisation und höhere Behandlungskosten.

Die aktuell vorliegende Datenlage zeigt keine eindeutige Überlegenheit einer der beiden Strategien. Empfehlungen zu präoperativem Screening oder verlängerter Antibiotikaprophylaxe existieren bislang nicht [8]. Bisher publizierte Studien sind überwiegend retrospektiv und weisen eine geringe Fallzahl auf. Hemmann et al. führten ein einzeitiges Vorgehen durch und beschrieben einen positiven Erregernachweis bei 10 % ihrer Fälle sowie eine Rate an periprothetischen Infektionen von 5,8 % [24]. Eine andere Studie verglich ein- und zweizeitige Operationsverfahren und fand periprothetische Infektionen ausschließlich in der Gruppe mit zweizeitigem Vorgehen (4,2 %), wobei die Infektion erst nach Implantation eines temporären Spacers auftrat. Die Autoren betonen, dass auch das zweizeitige Vorgehen selbst ein Risikofaktor für die Entstehung einer periprothetischen Infektion darstellen kann [18]. Scholten et al. konnten in ihrem Patientenkollektiv eine höhere Rate an periprothetischen Infektionen nach einzeitigem Vorgehen nachweisen (8,6 % vs. 3,8 %) und schlussfolgerten, dass ein zweizeitiges Vorgehen hinsichtlich einer Infektionskontrolle vorzuziehen sei [19]. Demgegenüber zeigten Klatte et al., dass das einzeitige Vorgehen sicher ist: Bei 122 ausgewerteten Fälle trat keine periprothetische Infektion auf [17]. Ähnliche Ergebnisse beschrieb La Camera in einer Match-Pair-Studie mit 127 Patienten, die ebenfalls keine erhöhten Infektionsraten im Vergleich zu primärer Hüft-TEP beobachteten ([8]; Tab. 1).

Die vorliegenden Arbeiten verdeutlichen die widersprüchliche Datenlage zu diesem Thema; der Vergleich zeigt teils erhebliche Unterschiede, sowohl hinsichtlich des Anteils nachgewiesener Erreger als auch in der Rate periprothetischer Infektionen. Als limitierende Faktoren sind hierbei insbesondere die unterschiedlichen diagnostischen Strategien sowie die Verwendung variierender Methoden zum Erregernachweis zu nennen. Dazu zählen die Anzahl der entnommenen Gewebeproben, die Durchführung einer Implantatsonikation und der Einsatz von PCR-Verfahren.

Positive mikrobiologische Befunde wurden bei mehr als der Hälfte der Patienten nach Entfernung von Osteosynthesematerial nachgewiesen, obwohl klinisch und paraklinisch kein Hinweis auf eine Infektion bestand [33]. George et al. fanden beim einliegenden Osteosynthesematerial im Rahmen eines einzeitigen Wechsels in 51 % der Fälle einen Erregernachweis [20]. Eine weitere Studie zeigte ebenfalls hohe Kolonisationsraten bei Materialentfernung, allerdings signifikant höher bei Implantaten im Kniegelenk als im Hüftgelenk (14 % vs. 2 %, *p* = 0,017) [34]. Als häufigste Erreger wurden Koagulase-negative Staphylokokken identifiziert (68,1 %) [34]. Bisher ist unklar, ob diese Befunde zwangsläufig mit einer erhöhten Rate an periprothetischen Infektionen einhergehen [35].

Ein Konsens zum Vorteil eines operativen Vorgehens fehlt bisher, daher ist eine Patientenselektion und Risikostratifizierung für eine individuelle Indikationsstellung entscheidend.

## Diagnostische Kriterien

### Hüftgelenkspunktion

Eine präoperative Hüftgelenkspunktion zur Diagnostik einer periprothetischen Infektion zeigt eine Sensitivität von 59–82 % und eine Spezifität von 94–100 % [36–38]. Da einliegendes Osteosynthesematerial nicht zwangsläufig Kontakt zum Hüftgelenk hat, liefert die Aspiration der Gelenkflüssigkeit keine verlässliche Aussage über eine mögliche Kolonisation des Osteosynthesematerials. Eine starke Korrelation zwischen präoperativer Aspiration und intraoperativen Kulturen konnte bisher nicht festgestellt werden [39].

### Infektionsparameter

Ein direkter Zusammenhang zwischen präoperativ erhöhtem C‑reaktiven Protein und dem Auftreten einer periprothetischen Infektion konnte bislang nicht eindeutig nachgewiesen werden [18, 24]. Cichos et al. schlugen einen CRP-Grenzwert < 12 mg/l als Kriterium für ein einzeitiges Vorgehen vor [40]. Während eine andere Arbeit erhöhte präoperative Infektionsparameter als Risikofaktor für eine periprothetische Infektion identifizierten [41], konnte in der Studie von Klatte et al. selbst bei erhöhten präoperativen Inflammationswerten kein Fall einer periprothetischen Infektion beobachtet werden. La Camera et al. führten die niedrige Infektionsrate unter anderem darauf zurück, dass in ihrer Kohorte keine Patienten mit erhöhten CRP-Werten eingeschlossen wurden [8].

### Frakturheilung

Das Ausbleiben einer Frakturheilung sowie eine Pseudarthrose kann Hinweis auf eine Infektion sein. In der Literatur finden sich zahlreiche Arbeiten, die aufzeigen, dass Pseudarthrosen trotz fehlender klinischer oder laborchemischer Infektzeichen intraoperativ positive mikrobiologische Befunde liefern können [42]. Solche unerwarteten Nachweise („surprise positive cultures“) sind allerdings in ihrer Häufigkeit sehr unterschiedlich beschrieben. Diese Unterschiede sind vor allem auf die variierenden Definitionen einer vermeintlich aseptischen Pseudarthrose sowie auf institutionell unterschiedliche Strategien zur Probenentnahme, Auswertung und Behandlung zurückzuführen [43, 44]. Scholten et al. beschrieben eine höhere Inzidenz von periprothetischen Infektionen bei einzeitigem Vorgehen aufgrund einer höheren Anzahl von Fällen mit ausbleibender Frakturheilung in dieser Gruppe [19]. Demgegenüber sahen andere Arbeiten, trotz eingeschlossener Fälle mit ausbleibender Frakturheilung, keine erhöhte Rate an periprothetischer Infektion [9, 17].

## Algorithmus der eigenen Klinik

**Abb. 1:**
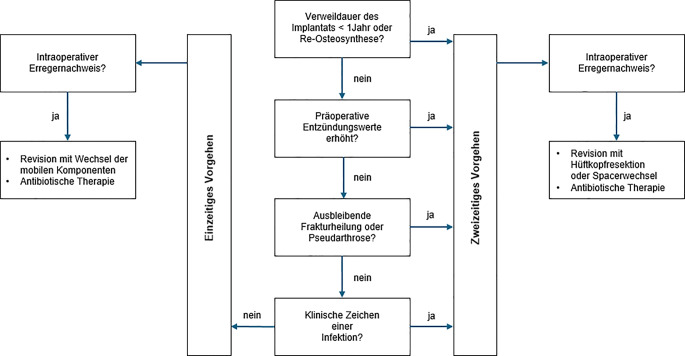
Klinischer Entscheidungsalgorithmus zur Konversions-Hüfttotalendoprothese (K-HTEP) nach Osteosyntheseversagen proximaler Femurfrakturen in unserer Klinik. Der Algorithmus berücksichtigt klinische Infektzeichen, laborchemische Parameter, Frakturheilung und Verweildauer des Implantats. Je nach Befund wird ein einzeitiges oder zweizeitiges Vorgehen gewählt. Intraoperative mikrobiologische Diagnostik (Gewebeproben, Sonikation, PCR) dient der Infektionskontrolle und der weiteren Therapieplanung

In unserer Klinik wird ein Entscheidungsalgorithmus zur K‑HTEP angewendet, der klinische Infektzeichen, laborchemische und radiologische Befunde sowie die Frakturheilung berücksichtigt (Abb. 1).

Voraussetzung für ein *einzeitiges Vorgehen* ist das Fehlen von Osteosyntheseversagen oder nicht konsolidierter Fraktur/Pseudarthrose. Zusätzlich müssen die präoperativen Entzündungsparameter im Normbereich liegen und der klinische Befund unauffällig sein. Bei diesen Patienten kann die Hüft-TEP direkt implantiert werden, ohne dass ein temporärer Spacer erforderlich ist (Fallbeispiel 1).

Ein *zweizeitiges Vorgehen* wird in allen anderen Fällen durchgeführt, z. B. bei ausbleibender Frakturheilung/Pseudarthrose oder auffälligen Entzündungsparametern. Zunächst wird das Osteosynthesematerial entfernt, ggf. der Hüftkopf reseziert, und ein antibiotikabeladener Spacer eingesetzt. Nach ausgeheilter Infektion erfolgt die definitive Implantation der Hüft-TEP (Fallbeispiel 2).

### Intraoperative Diagnostik

Standardmäßig werden in unserer Klinik fünf Gewebeproben für die Mikrobiologie entnommen. Das Osteosynthesematerial wird zur Sonikation eingesandt und zusätzlich eine PCR-Diagnostik durchgeführt.

### Vorgehen bei positivem Erregernachweis

Wird während der Entfernung des Osteosynthesematerials ein Erreger nachgewiesen, erfolgt eine 6 Wochen dauernde, gezielte antiinfektive Therapie. Beim zweizeitigen Vorgehen wird dabei der Spacer ggf. ausgetauscht oder neu eingebracht (Fallbeispiel 2). Beim einzeitigen Vorgehen führt ein positiver intraoperativer Befund zur Revision der mobilen Implantatteile, während gleichzeitig eine antiinfektive Therapie eingeleitet wird, die auch Biofilmstrukturen adressiert.

### Klinisches Fallbeispiel 1

**Abb. 2:**
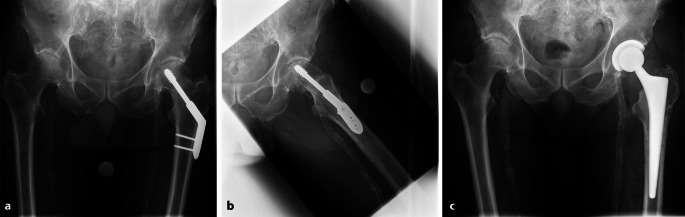
**a** und **b** Präoperative Röntgenaufnahmen zeigen eine sekundäre Koxarthrose bei einliegender dynamischer Hüftschraube links. Die Osteosynthese erfolgte 2 Jahre zuvor nach einer medialen Schenkelhalsfraktur. Klinischer und paraklinischer Befund waren unauffällig, sodass ein einzeitiges Vorgehen durchgeführt wurde. Intraoperative Proben und die Sonikation blieben steril. **c** Postoperatives Röntgenbild nach Hüfttotalendoprothesenimplantation links zeigt die regelrechte Lage der Endoprothese. Der postoperative Verlauf war komplikationslos und es traten keine Zeichen einer periprothetischen Infektion auf.

Die Vorstellung eines 67-jährigen Patienten in unserer Klinik erfolgte aufgrund einer sekundären Koxarthrose links. Zwei Jahre zuvor wurde eine Schenkelhalsfraktur mittels dynamischer Hüftschraube versorgt (Abb. 2a, b). Als relevante Nebendiagnosen bestanden eine pAVK Stadium III links, eine koronare 2‑Gefäßerkrankung mit 3‑facher Bypassoperation, arterielle Hypertonie, Hyperlipidämie, Nikotinanamnese sowie eine COPD mit Lungenemphysem.

Der klinische und paraklinische Befund war unauffällig: Leukozyten 8,7 × 10^9^/l, CRP 0,8 mg/l. Bei fehlenden Infektzeichen wurde ein einzeitiges Vorgehen gewählt. Es erfolgte die Materialentfernung und die Implantation einer zementfreien Hüfttotalendoprothese in einer Operation. Intraoperativ konnte kein Erregernachweis erbracht werden. Der postoperative Verlauf war komplikationslos, insbesondere zeigten sich keine Anzeichen einer periprothetischen Infektion (Abb. 2c).

### Klinisches Fallbeispiel 2

**Abb. 3:**
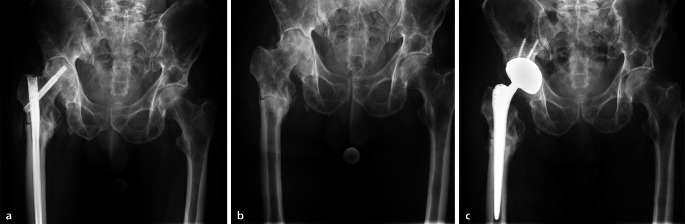
**a** Präoperatives Röntgenbild zeigt eine sekundäre Koxarthrose rechts mit einliegendem proximalem Femurnagel. Die Marknagelosteosynthese erfolgte nach einer subtrochantären Femurfraktur zwei Jahre zuvor. Die präoperative Labordiagnostik zeigte normwertige Entzündungswerte. Der klinische Befund war unauffällig. Es zeigte sich jedoch eine Pseudarthrose, sodass die Indikation zum zweizeitigen Vorgehen gestellt wurde. **b** Röntgenaufnahme nach Entfernung des proximalen Femurnagels rechts. Intraoperative Proben und die Sonikation ergaben den Nachweis von Staphylococcus epidermidis. Es erfolgte eine Revisionsoperation mit Hüftkopfresektion und Spacer-Implantation, ergänzt durch eine resistenzgerechte antiinfektive Therapie. **c** Postoperative Röntgenaufnahme nach Implantation der Hüfttotalendoprothese rechts zeigt einen regelrechten Befund. Die intraoperativen Proben blieben steril. Postoperative Komplikationen, insbesondere eine periprothetische Infektion, traten nicht auf.

Ein 72-jähriger Patient stellte sich in unserer Klinik mit einer sekundären Koxarthrose rechts vor. Zwei Jahre zuvor erfolgte bei einer subtrochantären Femurfraktur eine Marknagelosteosynthese. Als Nebenerkrankungen bestand ein multiples Myelom. In der präoperativen Labordiagnostik waren die Entzündungsparameter mit Leukozyten von 4,5 × 10^9^/l und einem CRP von 3,5 mg/l normwertig. Der klinische Lokalbefund war ebenfalls unauffällig. In der Röntgendiagnostik zeigte sich jedoch eine Pseudarthrose, sodass entsprechend des internen Behandlungsalgorithmus ein zweizeitiges Vorgehen gewählt wurde (Abb. 3a). Im ersten Schritt erfolgte die Entfernung des Marknagels sowie die Entnahme multipler Gewebeproben. Zusätzlich wurde das explantierte Material zur Sonikation eingesendet (Abb. 3b).

Intraoperativ zeigte sich in sämtlichen entnommenen Gewebeproben sowie in der Materialsonikation der Nachweis von Staphylococcus epidermidis. Daraufhin erfolgte die Revision mit Hüftkopfresektion und Einlage eines temporären Antibiotika-Spacers. Ergänzend wurde eine resistenzgerechte antiinfektive Therapie eingeleitet und für 6 Wochen fortgeführt.

Nach Ausheilung der Infektion wurde im zweiten Schritt eine zementfreie Hüfttotalendoprothese implantiert (Abb. 3c). Die intraoperativen Proben blieben steril. Der postoperative Verlauf war komplikationslos, insbesondere traten keine Zeichen einer periprothetischen Infektion auf.

## Fazit für die Praxis

- Die Konversions-Hüfttotalendoprothese nach Osteosyntheseversagen proximaler Femurfrakturen bleibt ein komplexes Verfahren.
- Ein einzeitiges Vorgehen ist bei Patienten mit konsolidierter Fraktur, normwertigen Laborwerten und unauffälligem klinischen Befund möglich.
- Ein zweizeitiges Vorgehen empfiehlt sich bei ausbleibender Frakturheilung, auffälligen Laborwerten oder klinischen Befunden sowie bei kurzer Implantatverweildauer (< 1 Jahr).
- Eine standardisierte intraoperative mikrobiologische Diagnostik unterstützt die Optimierung der Behandlung.
- Prospektive, multizentrische Studien sind notwendig, um zukünftig belastbare, evidenzbasierte Empfehlungen ableiten zu können.

## Abkürzungen

CRP: C-reaktives Protein
K-HTEP: Konversions-Hüfttotalendoprothese
PCR: Polymerase Chain Reaction
